# Real-time AI-guided ultrasound localization method for breast tumor rotational resection

**DOI:** 10.3389/fonc.2025.1675180

**Published:** 2025-10-24

**Authors:** Hang Sun, Hongjie Zhu, Menghan Zhang, Hong Li, Xinran Shao, Yunzhi Shen, Pingdong Sun, Jing Li, Jizhou Yang, Lei Chen, Jianchun Cui

**Affiliations:** ^1^ School of Information Science and Engineering, Shenyang Ligong University, Shenyang, China; ^2^ College of Medicine and Biological Information Engineering, Northeastern University, Shenyang, China; ^3^ Department of Thyroid and Breast Surgery, Liaoning Provincial People’s Hospital (People’s Hospital of China Medical University), Shenyang, China; ^4^ China Medical University, Shenyang, China; ^5^ Dalian Medical University, Dalian, China; ^6^ Liaoyang County Hospital, Liaoyang, China; ^7^ Department of Radiology, Affiliated Hospital of Guizhou Medical University, Guiyang, China

**Keywords:** breast tumor, deep learning, real-time positioning, minimally invasive rotational resection, ultrasound guidance

## Abstract

**Introduction:**

Breast tumors, predominantly benign, are a global health concern affecting women. Vacuum-assisted biopsysystems (VABB) guided by ultrasound are widely used forminimally invasive resection, but their reliance on surgeon experience and positioning challenges hinder adoption in primary healthcare settings. Existing AI solutions often focus on static ultrasound image analysis, failing to meet real-time surgical demands.

**Methods:**

This study presents a real-time positioning system for breast tumor rotational resection based on an optimized YOLOv11n architecture to enhance surgical navigation accuracy. Ultrasound video data from 167 patients (116 for training, 33 for validation, and 18 for testing) were collected to train the model. The model’s architecture was optimized across three major components: backbone, neck, and detection head. Key innovations include integrating MobileNetV4 Inverted Residual Block and MobileNetV4 Universal Inverted Bottleneck Block to reduce model parameters and computational load while improving inference efficiency.

**Results:**

Compared with the baseline YOLOv11n, the optimized YOLOv11n+ model achieves a 17.1% reduction in parameters and a 27.0% reduction in FLOPS, increasing mAP50 for cutter slot and tumor detection by 2.1%. Two clinical positioning algorithms (Surgical Method 1 and Surgical Method 2) were developed to accommodate diverse surgical workflows. The system comprises a deep neural network for target recognition and a real-time visualization module, enabling millisecond-level tracking, precise annotation, and intelligent prompts for optimal resection timing.

**Conclusion:**

These research findings provide technical support for minimally invasive breast tumor resection, holding the promise of reducing reliance on surgical experience and thereby facilitating the application of this technique in primary healthcare institutions.

## 1 Introduction

Breast tumors are among the most prevalent diseases in women worldwide, with a substantial proportion being benign (1, 2). The ultrasound-guided Vacuum-Assisted Biopsy System (VABB) has become the standard minimally invasive treatment for benign breast tumors, owing to its advantages of reduced trauma, rapid recovery, and precise localization (3). Since its introduction in 1994, VABB technology has undergone continuous improvements and is now also a vital tool in early breast cancer diagnosis (4, 5). However, its clinical effectiveness largely depends on surgeons’ expertise in real-time ultrasound image interpretation and precise instrument manipulation. This dependency limits the widespread implementation of VABB in primary healthcare settings.

Recent advancements in artificial intelligence (AI) have mainly concentrated on analyzing static ultrasound images (5–10) for the classification (7, 8, 11–14) and segmentation (15–19) of breast tumors. Notably, Li Y et al. (6) developed an intelligent scoring system that integrates tumor oxygen metabolism features using multimodal AI algorithms for assessing malignancy. Similarly, Vigil et al. (12) proposed a dual-modality deep learning model that combines ultrasound images with radiomic features to improve the classification of benign and malignant tumors. However, these methods do not fully meet the real-time accuracy needs required for minimally invasive rotational resection of breast tumors (4).

To overcome these limitations, this study presents an optimized YOLOv11 deep learning model for real-time localization of the biopsy slot and tumor during surgery. The model incorporates MobileNetV4 Inverted Residual Block (MIRB) and MobileNetV4 Universal Inverted Bottleneck Block (MUIB) in the Backbone networks, reducing parameters and FLOPS by 17.1% and 27.0%, respectively, compared to the baseline YOLOv11n, while improving mAP50 by 3.0%. Two surgical algorithms were designed to accommodate different clinical practices. The integrated system provides real-time tracking and visualization of the cutter slot and tumor, offering intelligent prompts for optimal resection timing.

These results demonstrate the potential of the proposed approach to enhance surgical precision and reduce reliance on surgeon experience, thereby facilitating the adoption of minimally invasive breast tumor rotational resection in primary healthcare institutions.

## 2 Materials and methods

### 2.1 Materials

In this study, the ultrasound video data of 167 patients who underwent vacuum-assisted minimally invasive rotational resection of breast tumors at Liaoning Provincial People’s Hospital from May 2023 to July 2024 were collected. Statistics on patient characteristics such as age distribution, tumor size range, and histopathological types are shown in **Table 1**. The ultrasound imaging systems used were the GE Logiq P3 and SonoScape E1 Exp. The vacuum-assisted tumor rotational resection system was EnCor^®^ (Model: DR-ENCOR) produced by Bard in the United States, and the diameter of the rotational resection probe was 7G (Model: ECP017).

**Table 1:** Patient demographics.

Characteristic	Dataset
Age (years)	44 (34.5-50) ^a^
<40	67
40-49	56
49>	44
Tumor (mm)	10 (8-15) ^a^
<10	69
10—19	76
>19	22
Operation time (min)	10 (6-18) ^a^
BI-RADS	
3	66(39.52%)
4a	101 (60.48%)
Histopathological types	
Malignant	2
Inflammatory	15
Fibroadenosis	33
Fibroadenoma	117

a Median (interquartile range).

### 2.2 Data processing

To acquire effective training data, this study first performed screening and extraction of surgical video clips. Guided by professional surgeons, the original ultrasound surgical videos were systematically sorted and clipped. The extraction scope covered the complete operational process from the moment the rotational resection knife penetrated the breast tissue surface to the activation of the cutter slot for tumor resection. The video data during this period accurately captured the surgical positioning process, preserving not only the dynamic trajectory changes of the cutter slot and tumor location but also critical operational details such as the operator’s adjustment of instrument angle and depth. This fully meets the experimental requirements for real-time positioning of the cutter slot and tumor, as well as determination of the optimal resection site.

The study then proceeds to the key frame extraction phase. To balance data integrity with computational efficiency, a uniform sampling strategy is employed, extracting one frame every 10 seconds. This approach systematically captures essential surgical dynamics, such as the trajectory of the cutter slot and the morphological changes of the tumor. Additionally, it minimizes data redundancy that can occur with higher frequency sampling, which significantly lowers the computational costs associated with data storage and model training. This results in a time-efficient and representative image dataset for the accurate positioning of the cutter slot and the tumor in subsequent steps.

The image annotation process begins with a dual-review mechanism to ensure the accuracy of the annotated data. First, specialized doctors with over five years of clinical experience identify the locations of tumors and the cutter slot in each keyframe image, utilizing their professional medical knowledge. Next, experts with more than twenty years of clinical experience review and confirm the annotations. This hierarchical review process effectively reduces annotation errors, providing high-precision and reliable labeled data for training deep learning models and establishing a solid foundation for subsequent work.

At the same time, data augmentation techniques (20–26) such as color enhancement, mosaic enhancement, horizontal flipping, and scaling are comprehensively employed to simulate diverse surgical environments and changes in the quality of ultrasound images (**Table 2**), effectively improving the robustness and detection accuracy of the model in complex clinical scenarios.

**Table 2:** Data augmentation parameters.

Augmentation	Key	Range/Prob
Random crop fraction	crop_fraction	1
Rotation (°)	degrees	0
Random erasing prob.	erasing	0.4
Horizontal flip prob.	fliplr	0.5
HSV Hue gain	hsv_h	0.015
HSV Saturation gain	hsv_s	0.7
HSV Value gain	hsv_v	0.4
Mosaic prob.	mosaic	0.5
Scaling (gain)	scale	0.2

The study utilized a multicenter dataset consisting of 11,610 images from 167 cases. These cases were divided into training (116 cases), validation (33 cases), and test sets (18 cases) using a 7:2:1 ratio. This division strategy encompasses a diverse array of tumor morphologies, locations, and surgical scenarios, allowing for a comprehensive assessment of the model’s robustness and reliability in practical applications. Ultimately, this approach significantly enhances the model’s generalization ability.

### 2.3 Optimize network architecture

**Figure 1:**
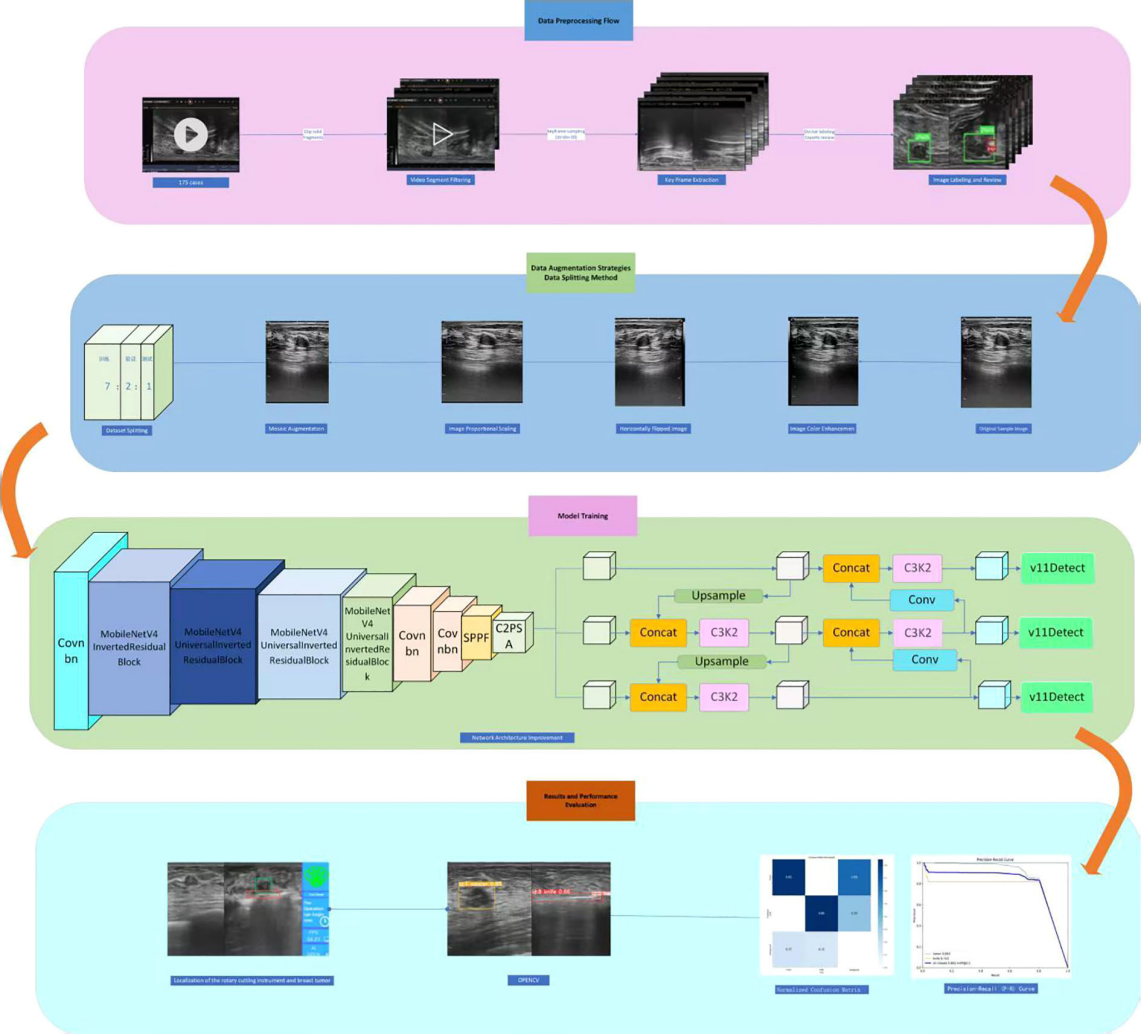
Methodological workflow.

Building upon the high-quality annotated dataset constructed in the preliminary stage, this study focuses on the requirements for real-time localization of cutter slot and tumors in the context of breast rotational resection. It systematically compares and analyzes different versions of the YOLO network architecture (27, 28). By comprehensively applying technical means such as model lightweight and optimized training methods, an efficient and precise real-time localization method is proposed. To clearly present the research context and technical roadmAP, the complete experimental design process has been sorted out and depicted in **Figure 1**, covering core aspects such as data processing, model construction, optimization strategies, and performance evaluation.

The clinical practice of breast rotational resection requires two critical factors for real-time ultrasound image processing: high inference speed and precise recognition accuracy. The traditional YOLOv11 model (29) has a complex network structure that consumes substantial computational resources and exhibits high inference latency, rendering it unsuitable for rapid ultrasound image analysis during surgeries on edge computing devices. To address these challenges, this study focuses on optimizing the YOLOv11 architecture. A key improvement involves incorporating MIRB and MUIB into the Backbone of YOLOv11. This modification significantly enhances the model’s performance across three dimensions: the Backbone, Neck, and Head. While markedly reducing the number of model parameters, it effectively maintains detection accuracy, achieving a balance between low latency and high precision. This optimization provides a reliable technical solution for real-time intraoperative localization.

In the Backbone of the model, ConvBN serves as the primary unit for feature extraction, providing a stable foundation for this process. To address the computational limitations of traditional architectures and enhance model efficiency, we introduce the MIRB and MUIB (30–33). The MIRB, which is based on depthwise separable convolution, is designed specifically for low-level feature extraction. Its design incorporates small channel numbers and shorter stride lengths, ensuring effective feature extraction while significantly lowering computational costs. On the other hand, the MUIB combines depthwise and expanded convolutions, focusing on mid-to-high-level feature extraction. By optimizing the computational workflow, these blocks effectively reduce model parameters while preserving detection accuracy, thereby improving overall computational efficiency.

A channel-increasing strategy with values of 40-80-128-480–512 is adopted to capture multi-scale image features from small to large. Meanwhile, 3×3 and 5×5 convolutional kernels are flexibly employed to fully leverage their advantages in local feature extraction. Additionally, an initial downsampling operation with a stride of 2 is performed to rapidly compress the feature mAP size, alleviating computational pressure for subsequent processing.

The Neck network enhances the model’s capability to detect targets of varying scales by integrating low-resolution and high-resolution features through a multi-level upsampling and feature fusion mechanism. To further boost inference efficiency, the lightweight C3K2 module is incorporated, which employs a local information pruning strategy to eliminate redundant computations. This approach accelerates the inference process while preserving detection accuracy, striking a favorable balance between detection speed and precision.

**Figure 2:**
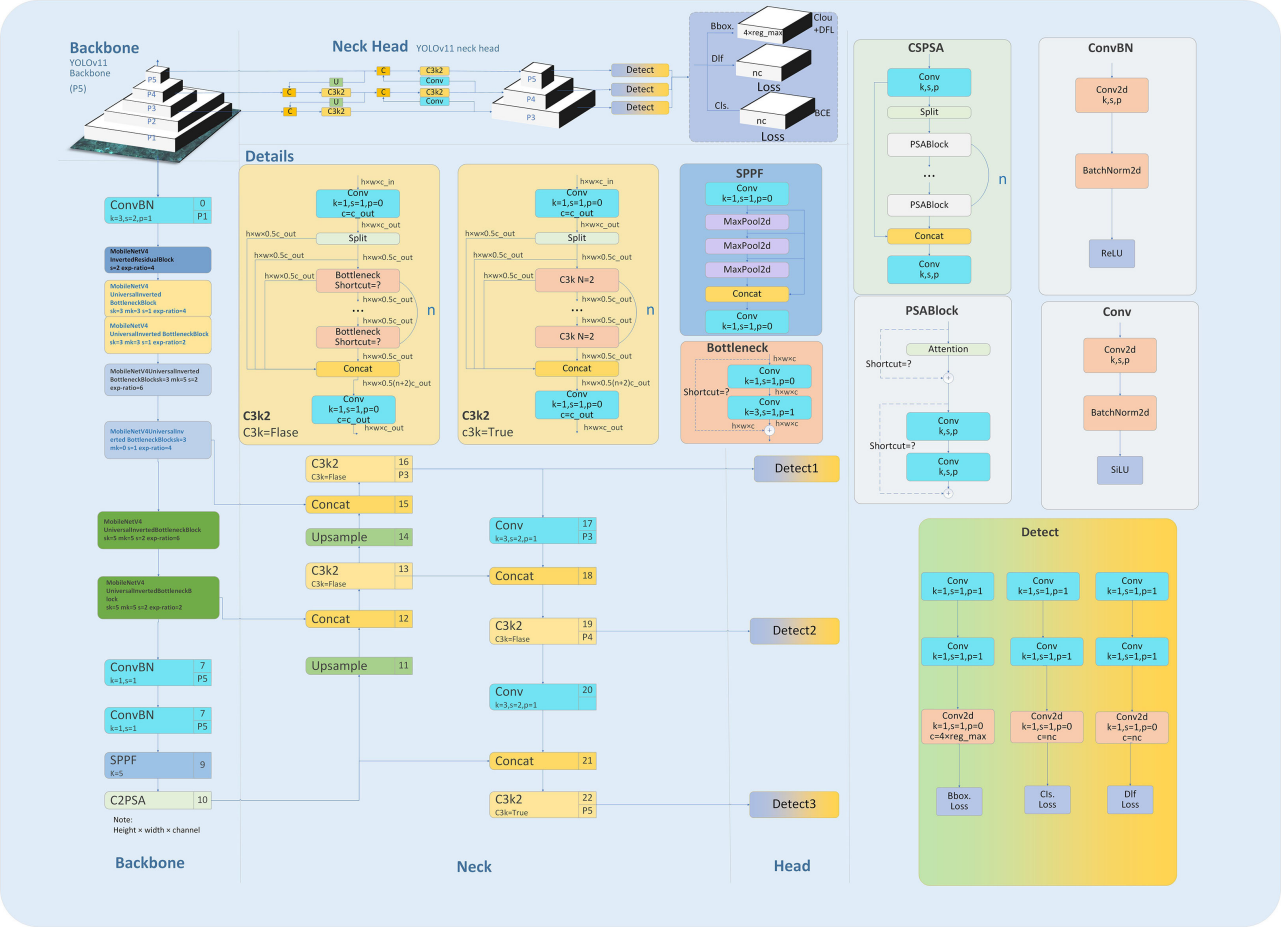
YOLOv11n+ architecture.

In summary, by integrating the ConvBN, MIRB module, MUIB module, SPPF module, C3K2 module, and cross-stage partial connection technology (34–37) (**Figure 2**), the optimized YOLOv11n and YOLOv11s architectures are designated as YOLOv11n+ and YOLOv11s+, respectively. As shown in the model parameter comparison in **Table 3**, each module exhibits clear functional specialization and efficient collaboration, demonstrating exceptional performance in small-target detection tasks.

**Table 3:** Comparison of original parameters and improved parameters.

Module	Original parameters	Improved parameters
MIRB	None	Output channels 80, 128; Kernel sizes 3×3, 5×5; Expansion ratios 2, 4, 6; Strides 1, 2
MUIB	None	Output channels 80, 160; Kernel sizes 3×3, 5×5; Expansion ratios 4, 6
SPPF	Output channels 1024; Kernel size 5×5	Output channels 80, 160; Kernel sizes 3×3, 5×5; Expansion ratios 4, 6
C3K2	Output channels 256, 512; No pruning	Output channels 256, 512; Pruning enabled (True)

### 2.4 Determination of cutter activation

This study addresses the core needs of breast rotational resection by developing an intelligent algorithm for accurately prompting the cutter slot of the rotational resection device to reach the specified position. Given the substantial variability in clinicians’ manipulation techniques when operating the rotational resection device and ultrasound probe, two differentiated implementation schemes (Procedure I and Procedure II) are designed to accommodate diverse surgical operation habits.

When the user selects Procedure I, the rotational resection cutter should be positioned beneath the target tumor to ensure both the tumor and cutter slot are within the ultrasound field of view. In this mode, the ultrasound probe must remain stable above the corresponding skin area to maintain image stability and avoid significant movement. Once activated, the algorithm monitors the real-time spatial relationship between the cutter slot and tumor. The cutter slot is considered correctly positioned for activation when both appear in the image, with the slot horizontally located to the left of the tumor and vertically overlapping with it. Using the following parameters: Cutter slot: top-left coordinates (x1, y1), length L1, height H1; tumor: top-left coordinates (x2, y2), length L2, height H2. The specific positioning logic is categorized into three scenarios based on geometric dimensions:

a. Cutter slot larger than tumor (L1 - L2 > 20 pixels):

• Horizontal condition: x1 - L1 × 0.2 < x2

• Vertical condition: y2 - H2 × 1.1 < y1

Rationale: Ensures the slot horizontally covers the tumor’s left side and vertically overlaps with it at activation.

b. Cutter slot smaller than tumor (L2 - L1 > 20 pixels):

• Horizontal condition: x2 - L2 × 0.2 < x1

• Vertical condition: y2 - H2 × 1.1 < y1

Rationale: Reverse coordinate comparison ensures the tumor falls within the cutter’s horizontal range.

c. Similar sizes (|L1 - L2| < 20 pixels):

• Temporarily extend the tumor’s length by 20% (L2’ = L2 × 1.2) while keeping (x2, y2) unchanged.

• Apply scenario (b) rules to standardize calculations and ensure compatibility across size variations.

This adaptive approach enables precise positioning guidance tailored to diverse anatomical structures and equipment specifications.

In Procedure II, the user positions the rotational resection cutter laterally to the tumor. This positioning requires the ultrasound probe to switch back and forth between the cutter and the skin over the tumor, resulting in the two appearing alternately in the ultrasound’s field of view.

The system begins by analyzing multi-frame images to accurately document the spatial position of the cutter slot while continuously tracking the real-time position of the tumor. Using the same spatial positioning algorithm as in Procedure I, the system dynamically calculates the coordinates and size relationships between the cutter slot and the tumor.

When the cumulative number of frames detecting positional overlap between the cutter and the tumor reaches 500, the system activates a prompt mechanism to notify the user that the cutter is in the optimal resection position. This provides a reliable basis for making decisions regarding subsequent surgical operations.

## 3 Results

### 3.1 Model training results

The experiments were conducted on an NVIDIA RTX 4090 GPU using the PyTorch 2.6.0 framework, with multiple strategies employed to ensure training effectiveness. A training schedule of 333 epochs was combined with an early stopping mechanism (terminating training if validation set performance did not improve for 55 consecutive epochs) to prevent overfitting (38, 39). The performance of various optimizers was compared in the experiment (**Table 4**). The Stochastic Gradient Descent (SGD) optimizer with momentum was selected, and its initial learning rate was set to 0.01 to ensure stable model convergence (40). The batch size was set to 64 to balance computational efficiency and training stability.

**Table 4:** Comparative analysis of optimizer.

Architecture	Adam	AdamW	NAdam	SGD
Precision (tumor)	0.861	0.912	0.854	0.862
Recall (tumor)	0.78	0.743	0.761	0.725
mAP50 (tumor)	0.868	0.823	0.855	0.827
Specificity (tumor)	0.845	0.831	0.815	0.834
MCC (tumor)	0.622	0.531	0.568	0.55
ERR (tumor)	0.191	0.231	0.217	0.23
Precision (cutter slot)	0.738	0.809	0.792	0.850
Recall (cutter slot)	0.575	0.722	0.784	0.746
mAP50 (cutter slot)	0.651	0.756	0.608	0.799
Specificity (cutter slot)	0.876	0.759	0.786	0.799
MCC (cutter slot)	0.480	0.474	0.571	0.534
ERR (cutter slot)	0.238	0.263	0.215	0.233
Precision (all)	0.799	0.861	0.823	0.856
Recall (all)	0.677	0.732	0.772	0.736
mAP50 (all)	0.760	0.789	0.732	0.813
Specificity (all)	0.861	0.795	0.800	0.817
MCC (all)	0.551	0.510	0.571	0.542
ERR (all)	0.222	0.245	0.215	0.232

*p < 0.05.

**Figure 3:**
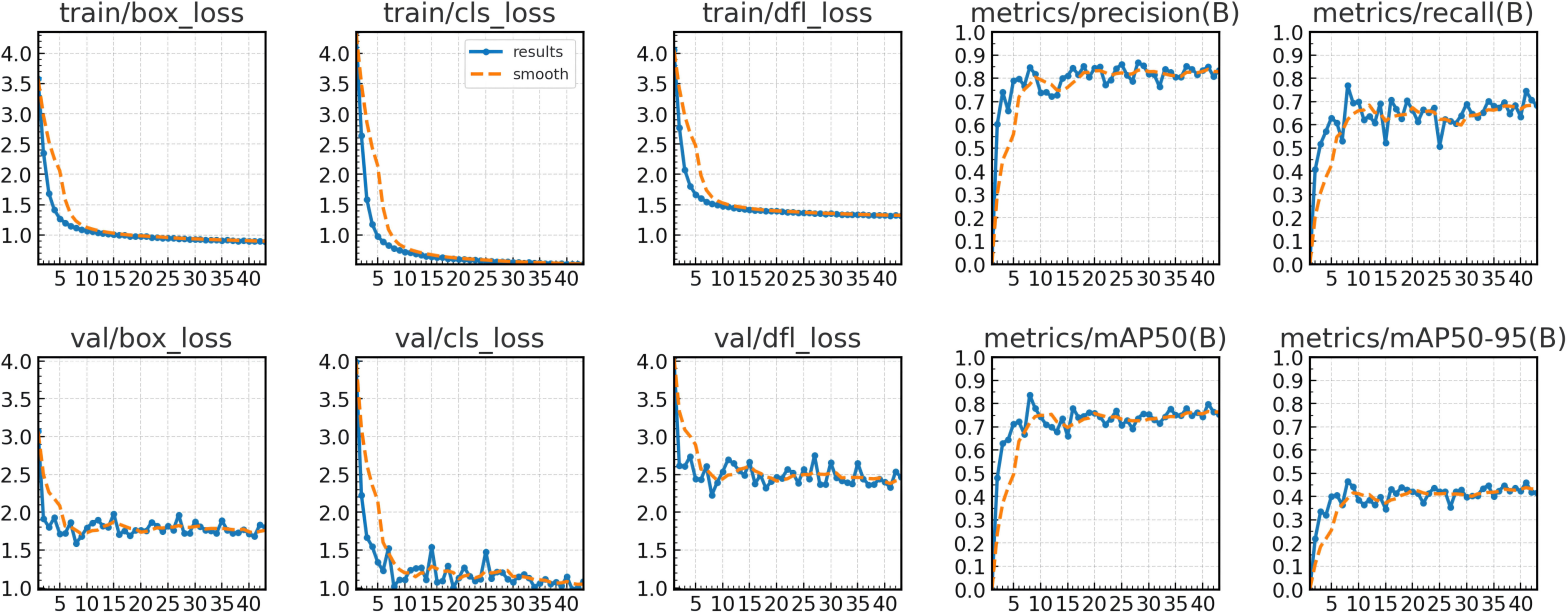
Training and validation loss (box_loss, cls_loss, dfl_loss) across 43 epochs and performance curves (precision, recall, mAP50, mAP50-95).

The training curves demonstrate effective model convergence within 35 epochs, with the detection losses (box_loss, cls_loss, dfl_loss) of both training and validation sets steadily decreasing, indicating robust learning without overfitting. The precision and recall on the training set showed continuous performance improvements, confirming the model’s optimized capabilities in both localization and classification tasks. **Figure 3** illustrates the relevant results over 35 training epochs.

The experiments systematically compared different YOLO architectures with our improved versions to quantitatively evaluate the impact of each proposed enhancement. Through controlled variable experiments, this study assessed key performance metrics, with results presented in **Table 5**. The proposed approach of integrating MIRB and MUIB into YOLOv11n+ achieves the best real-time detection performance for cutter slot and tumors.

**Table 5:** Performance metrics comparison.

Architecture	Total params	FLOPS	CPU latency	GPU latency
YOLOv8s	11126358	28.4G	34.2ms	1.2ms
YOLOv11s	9413574	21.3G	32.6ms	1.2ms
YOLOv11n	2582542	6.3G	12.6ms	0.7ms
YOLOv11s+(Ours)	7477470	13.9G	28.5ms	1.1ms
YOLOv11n+(Ours)	2140390	4.6G	12.2ms	0.7ms

The YOLOv11n+ model has a parameter count of 2,140,390, the smallest among all models, indicating its highly compact design, which helps reduce storage requirements and improve deployment flexibility. Secondly, the model’s FLOPS is 4.6G, far lower than other models, meaning it requires fewer computational resources for image processing, thereby reducing energy consumption and hardware costs. In terms of inference speed, the YOLOv11n+ model also performs excellently. Its CPU inference time is only 12.2 ms, and GPU inference time is 0.7 ms—both metrics are the lowest among all models. This demonstrates that the YOLOv11n+ model can provide faster response speeds in practical applications, which is particularly critical for real-time processing scenarios.

### 3.2 Detection of cutter slot and tumor

Building upon the optimized network model described above, the experiments systematically compared the precision, recall, and mAP50 values of different YOLOv architectures for cutter slot, tumors, and their combined detection, as shown in **Table 6**.

**Table 6:** Comparative analysis of model performance.

Architecture	YOLOv8s	YOLOv11s	YOLOv11n	YOLOv11s+(Ours)	YOLOv11n+(Ours)
Precision (tumor)	0.827	0.820	0.830	0.815	0.862
Recall (tumor)	0.759	0.758	0.760	0.759	0.725
mAP50 (tumor)	0.821	0.823	0.835	0.835	0.827
Specificity (tumor)	0.821	0.806	0.826	0.815	0.834
MCC (tumor)	0.579	0.562	0.585	0.574	0.55
ERR (tumor)	0.212	0.220	0.209	0.214	0.23
Precision (cutter slot)	0.801	0.809	0.783	0.798	0.850
Recall (cutter slot)	0.686	0.722	0.709	0.666	0.746
mAP50 (cutter slot)	0.735	0.756	0.742	0.724	0.799
Specificity (cutter slot)	0.845	0.789	0.736	0.765	0.799
MCC (cutter slot)	0.540	0.508	0.441	0.425	0.534
ERR (cutter slot)	0.231	0.248	0.279	0.293	0.233
Precision (all)	0.811	0.814	0.807	0.807	0.856
Recall (all)	0.722	0.740	0.734	0.713	0.736
mAP50 (all)	0.778	0.789	0.789	0.779	0.813
Specificity (all)	0.833	0.798	0.781	0.790	0.817
MCC (all)	0.559	0.535	0.513	0.500	0.542
ERR (all)	0.221	0.234	0.244	0.253	0.232

*p < 0.05.

As seen in **Table 4**, YOLOv11n demonstrates superior performance in tumor recognition compared to the proposed YOLOv11n+ model. However, when it comes to recognizing cutter slot and overall recognition performance, the optimized YOLOv11n+ model presented in this paper shows improved results. Based on the model training parameters and performance evaluation metrics outlined in **Table 3**, the YOLOv11n+ model not only significantly reduces the number of parameters for real-time inference but also enhances the recognition performance for both cutter slot and tumors.

**Figure 4:**
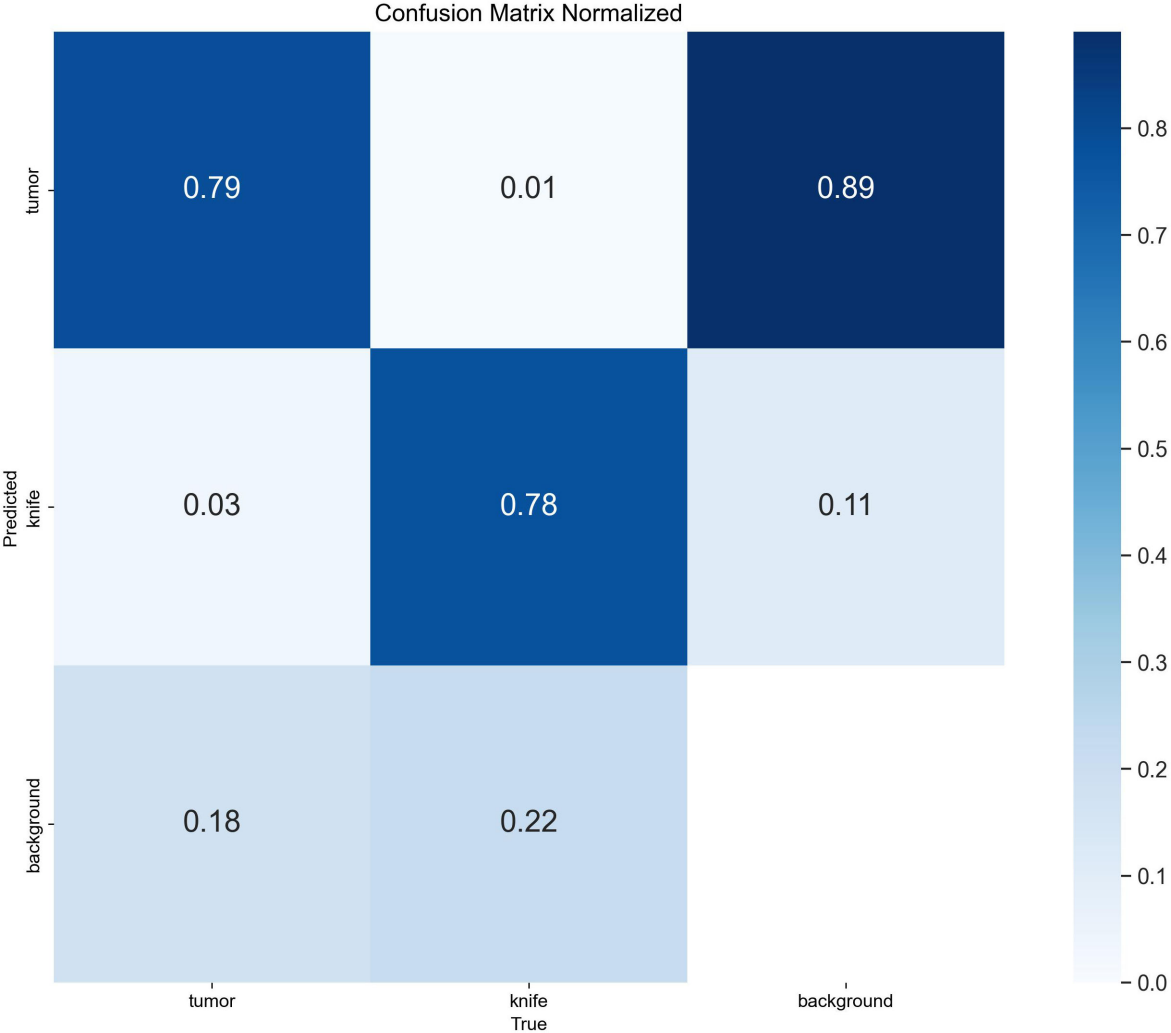
Normalized confusion matrix.

**Figure 5:**
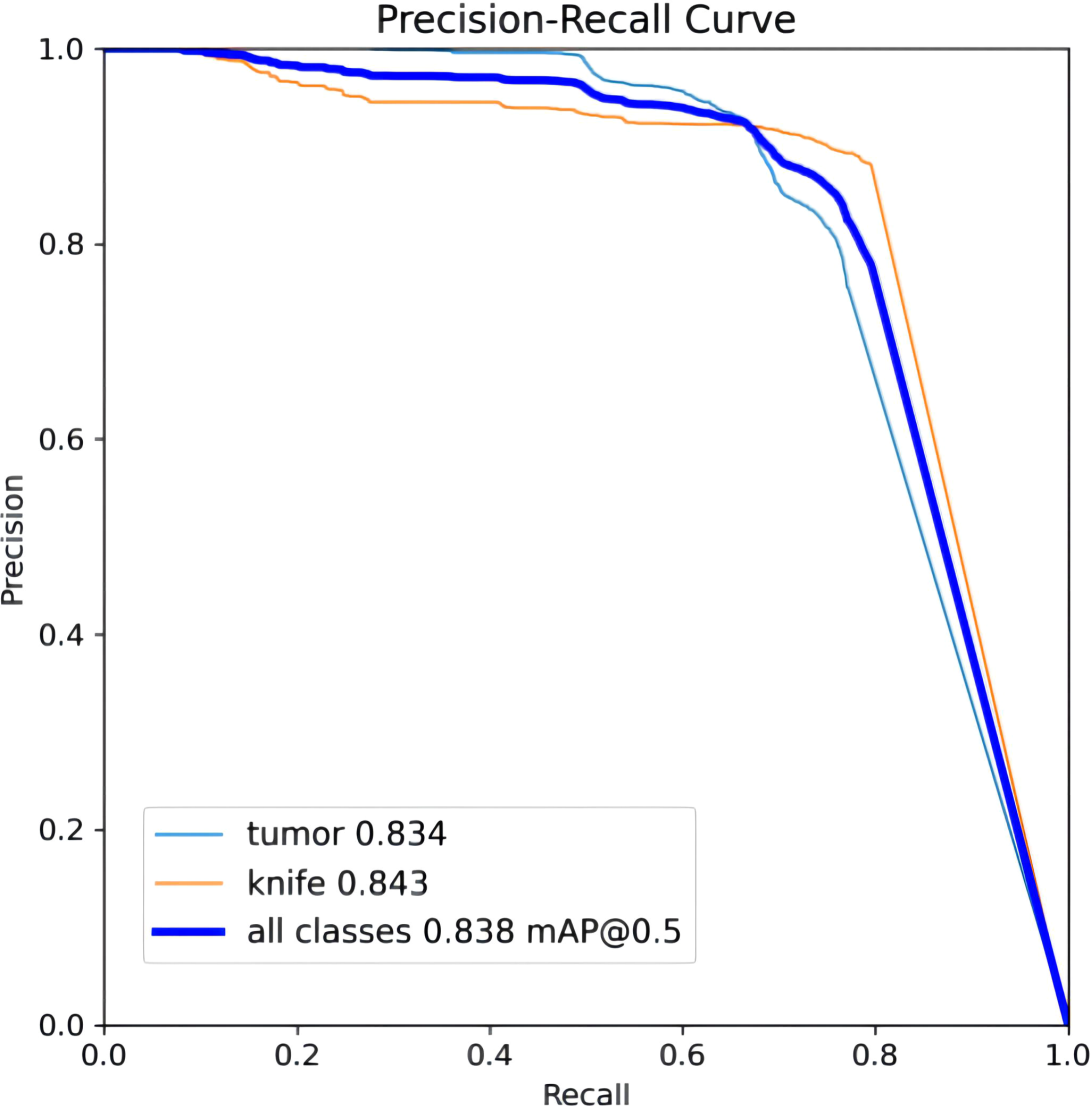
Precision-recall (P-R) curve.

To ensure a scientifically robust and reliable evaluation of model performance, this experiment employed a three-fold cross-validation strategy. Initially, all eligible cases were stratified such that each individual patient constituted a distinct unit for data partitioning. This approach was specifically designed to prevent data leakage by ensuring that samples originating from the same patient were not distributed across different folds. The quantitative evaluation outcomes are detailed in **Table 7**. **Figure 4** shows the normalized confusion matrix, while **Figures 5** **, 6** present the Precision-Recall (P-R) curve and ROC curve, both demonstrating that the proposed YOLOv11n+ network architecture exhibits excellent real-time recognition performance.

**Table 7:** Three-fold results of YOLOv11n+.

Fold	1	2	3	Avg
Precision (tumor)	0.803	0.911	0.873	0.862
Recall (tumor)	0.759	0.692	0.726	0.725
mAP50 (tumor)	0.833	0.821	0.829	0.827
Specificity (tumor)	0.741	0.906	0.853	0.834
MCC (tumor)	0.496	0.594	0.572	0.55
ERR (tumor)	0.248	0.218	0.221	0.23
Precision (cutter slot)	0.892	0.819	0.839	0.850
Recall (cutter slot)	0.776	0.722	0.741	0.746
mAP50 (cutter slot)	0.842	0.756	0.799	0.799
Specificity (cutter slot)	0.857	0.757	0.783	0.799
MCC (cutter slot)	0.62	0.469	0.514	0.534
ERR (cutter slot)	0.192	0.264	0.242	0.233
Precision (all)	0.847	0.865	0.856	0.856
Recall (all)	0.768	0.707	0.733	0.736
mAP50 (all)	0.838	0.789	0.814	0.813
Specificity (all)	0.799	0.831	0.818	0.817
MCC (all)	0.558	0.532	0.542	0.542
ERR (all)	0.22	0.241	0.231	0.232

**Figure 6:**
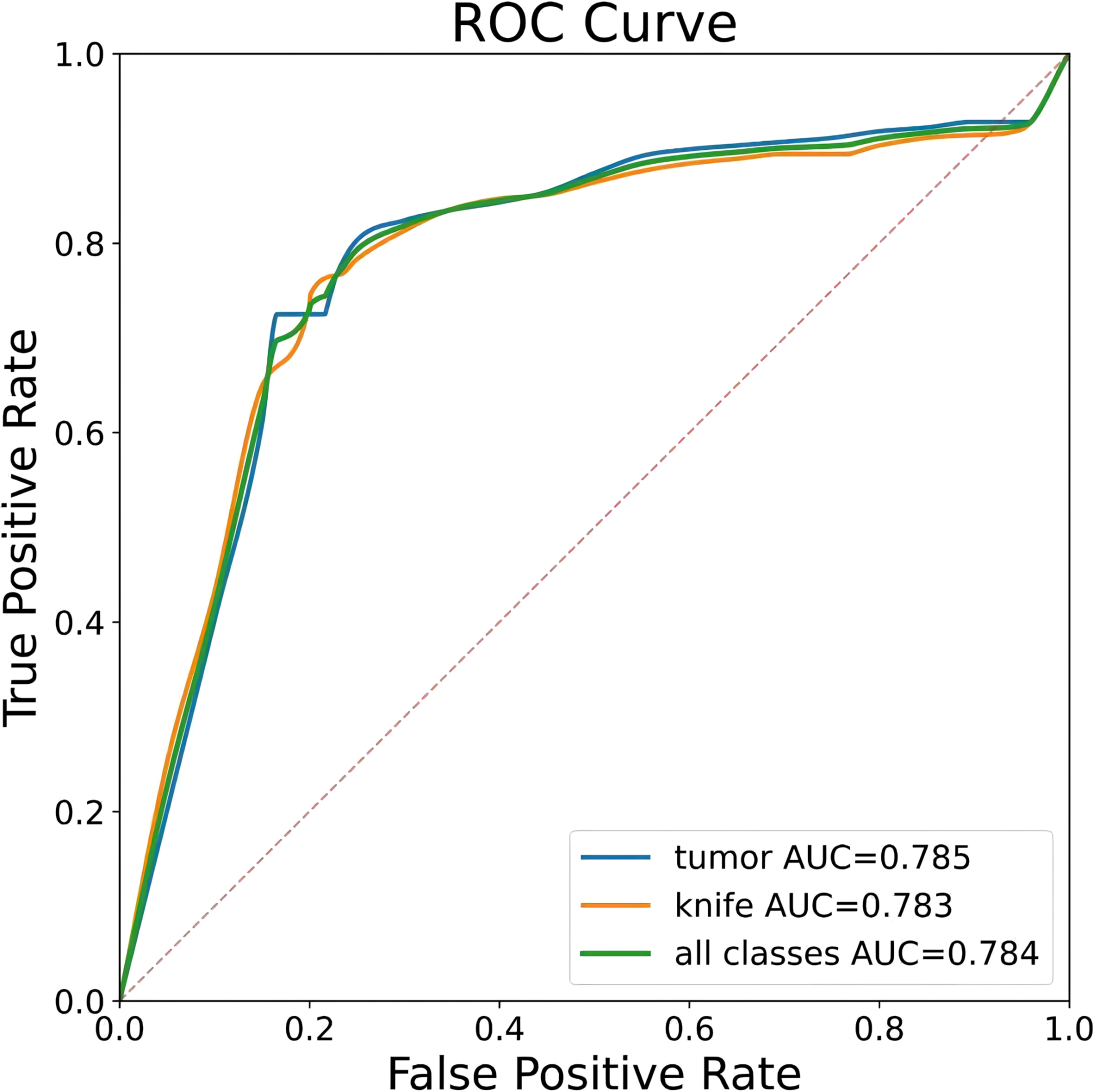
ROC curve.

From the Precision-Recall Curve in **Figure 5**, the recognition performance for the cutter slot, tumor, and their combined detection can be observed. When the recall rate for the cutter slot reaches approximately 0.75, the precision is around 0.85. In contrast, when the recall rate for the tumor reaches approximately 0.72, the precision remains at around 0.86, indicating that the model exhibits high precision and robustness in tumor recognition. Overall, the average performance of the combined detection still demonstrates that the optimized network model proposed in this paper has excellent recognition capabilities (41, 42).

### 3.3 Cutter slot activation command

The intelligent positioning system proposed in this paper comprises two core components: a deep neural network target recognition module and a real-time visualization module. The target recognition module is built based on the optimized YOLOv11n+ architecture, with structural improvements significantly enhancing recognition accuracy and computational efficiency, laying the foundation for intraoperative real-time guidance.

**Figure 7:**
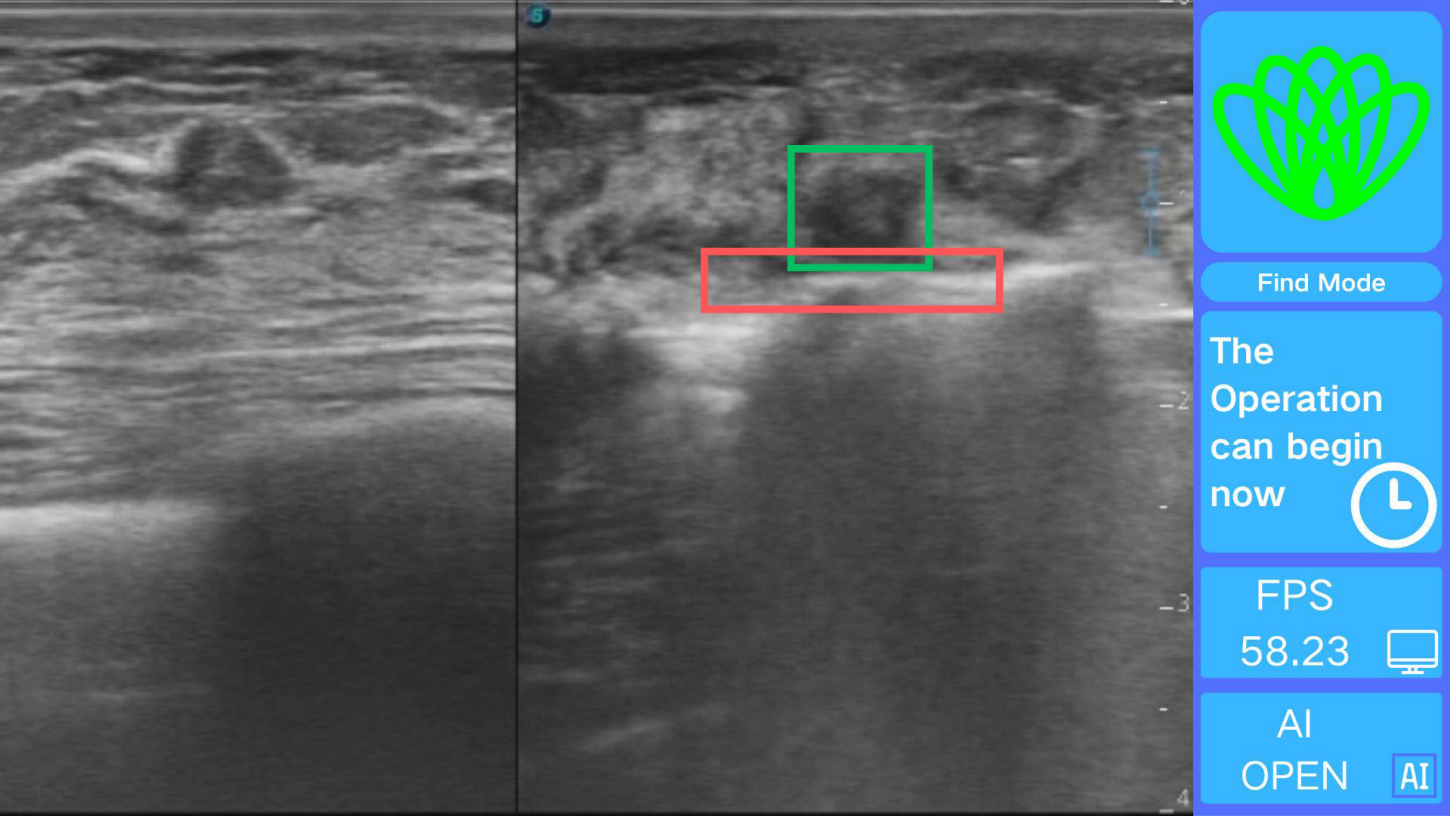
Real-time intelligent guidance interface demonstrating.

The real-time visualization module creates an interface using OpenCV 4.9.0 for processing real-time ultrasound video streams frame by frame. Each frame is analyzed quickly using the Intel OpenVINO 2025.0.0 toolkit, which adds visually distinct annotations. Based on the cutter slot activation determination method described earlier, a command stating, “The operation can begin now,” is issued, as illustrated in **Figure 7**.

The use of this system during surgery allows physicians to quickly and accurately determine the spatial relationship between the rotating cutter and the tumor, thereby reducing the overall procedure time. However, the positioning system does not directly decrease the incidence of surgical complications. The software can be easily installed on any standard computer and does not require high-end hardware. Its straightforward installation and user-friendly interface further ensure that clinicians can readily incorporate the system into their workflow.

## 4 Discussion

In the field of minimally invasive breast tumor treatment, the development of precise positioning technology is pivotal for enhancing surgical outcomes and patient prognosis. Addressing this critical need, this study innovatively introduces YOLOv11n+, an ultrasound-based positioning system tailored for minimally invasive breast tumor resection. Built upon the deeply optimized YOLOv11n+ model, the system enables real-time and accurate localization of both the cutter slot and tumors during surgery, offering surgeons intuitive and reliable surgical navigation support.

Compared to traditional YOLO architectures, the optimized YOLOv11n+ model achieves significant breakthroughs across multiple key performance metrics. In terms of detection accuracy, the model delivers real-time recognition precision of 0.850 for cutter slot and 0.862 for tumors, with corresponding recall rates of 0.746 and 0725. The mAP50 metrics reach 0.799 for cutter slot and 0.827 for tumors. These results fully demonstrate the YOLOv11n+ model’s capability for precise target recognition and localization in complex ultrasound imaging environments.

The improvement in model efficiency is a significant highlight of YOLOv11n+. Through in-depth architectural optimizations, the study introduced innovative elements, MIRB and MUIB, into the Backbone components. Compared to YOLOv11n, this strategic modification reduced the parameter count from 2,582,542 to 2,140,390 and decreased the FLOPS from 6.3G to 4.6G. In terms of inference speed, CPU processing time was shortened from 12.6 ms to 12.2 ms, while GPU inference achieved an impressive 0.7 ms—all while maintaining high real-time detection accuracy. This computational efficiency allows the YOLOv11n+ system to seamlessly manage real-time ultrasound video streams, providing robust technical support for immediate feedback during surgical procedures.

To comprehensively evaluate the performance of the proposed YOLOv11n+ and YOLOv11s+ models, we compared them with several state-of-the-art methods for breast lesion detection across diverse imaging modalities. As summarized in **Table 8**, FS-YOLOv9 achieved an mAP of 0.713 on MRI, indicating limited performance in this modality (14). YOLOv4, meanwhile, exhibited relatively low recall rates on digital mammography, highlighting suboptimal adaptability to dense breast tissues (43). Meng et al. proposed the DGANet model, which outperformed YOLOX and Faster R-CNN on a clinical dataset of 765 patients but suffered from relatively low precision (13). In contrast, our YOLOv11n+ models achieved mAP scores of 0.813 while maintaining a lightweight architecture and high inference efficiency, making them particularly well-suited for real-time intraoperative breast tumor excision.

**Table 8:** Comparative analysis with State-of-the-art methods.

Method	Data	Precision	Recall	mAP50
FS-YOLOv9	MRI	0.850	0.780	0.713
YOLOv4	Mammography	0.850	0.600	
DGANet	Ultrasound	0.762	0.841	0.831
YOLOv11n+ (ours)	Ultrasound	0.856	0.736	0.813

From a clinical application perspective, the real-time capability and high-precision positioning of the YOLOv11n+ model hold significant value. In minimally invasive breast tumor resection, accurately determining the positional relationship between tumors and cutter slot is critical for improving surgical success rates and reducing risks. The YOLOv11n+ model can continuously provide surgeons with accurate real-time feedback throughout the operation, assisting them in making more informed decisions. Additionally, the model’s low demand for computational resources enables it to operate stably in resource-constrained medical environments, significantly expanding its application scope and showing promise for widespread adoption in more grassroots medical facilities.

Technically, this study has notable strengths: (1) YOLOv11n+, with MIRB and MUIB integration, reduces parameters by 17.1% and FLOPS by 27.0% while boosting detection accuracy, achieving 0.7 ms GPU inference to meet real-time surgical needs; (2) Its dual-target detection (tumors and cutter slots) directly addresses VABB requirements, providing critical spatial feedback—outperforming static image-focused solutions; (3) The lightweight design (4.6G FLOPS) adapts to low-cost hardware, facilitating use in primary care.

Limitations include: a single-center dataset (167 patients) may restrict generalizability, requiring multi-center validation; performance is sensitive to ultrasound quality, with severe artifacts potentially degrading detection of small/irregular tumors; lack of direct comparison with latest real-time architectures (e.g., advanced Transformers); and reliance on 2D ultrasound limits depth information—integrating 3D or other modalities would need further optimization to balance precision and speed.

In summary, the positioning system built on the optimized YOLOv11n+ model has achieved remarkable advancements in recognition accuracy, real-time inference capability, and computational efficiency. These breakthroughs not only provide surgeons performing breast tumor resection with a powerful decision-making tool but also significantly enhance the safety and effectiveness of minimally invasive surgical techniques. By reducing computational complexity while enabling efficient on-device operation, the model fully demonstrates its immense potential in clinical applications. Looking ahead, this model will undergo rigorous multi-center clinical validation. However, there is currently a lack of prospective trial data. On the methodological front, we will explore additional deep learning models. Through continuous optimization and adaptive improvements, the system is designed to expand its applicability across various surgical scenarios, aiming to provide more accurate and safer medical services for a broader patient population.

## 5 Conclusion

The increasing incidence of breast tumors has resulted in a heightened demand for vacuum-assisted breast biopsy procedures. Successful execution of this technique necessitates physicians’ precise identification and localization of lesions via ultrasound imaging, presenting a significant technical barrier. This challenge particularly impedes the capacity of less-experienced practitioners and primary healthcare settings to perform such interventions effectively. This study developed the YOLOv11n+ real-time intelligent positioning system. Through deep architectural optimization, the system establishes an efficient visual interaction interface capable of millisecond-level dynamic tracking of the cutter slot and tumors during surgery, clearly presenting their spatial positional relationship through precise annotations. More critically, leveraging the deep neural network’s intelligent decision-making capabilities, the system can analyze the relative positions of surgical instruments and tumors. When the optimal operation timing is reached, it automatically generates high-precision prompt commands, explicitly instructing surgeons to activate the rotational cutter for tumor resection, thereby providing fully intelligent navigation support throughout the surgical procedure.

This study demonstrates significant application value as an innovative surgical assistive tool. Consequently, the research team plans to conduct multi-center clinical validation, further verifying the system’s safety, efficacy, and reliability through large-scale, multi-scenario testing with real-world cases. This effort aims to provide robust technical support for advancing minimally invasive surgery toward intelligent and precision-based approaches, facilitating the medical field’s pursuit of more efficient and safer diagnostic and therapeutic outcomes.

## Data Availability

The datasets presented in this article are not readily available because data usage must be restricted to researchers in the relevant field, and communication with the corresponding authors via email is required. Requests to access the datasets should be directed to Jianchun Cui, cjc7162003@aliyun.com; Hang Sun, sunhang84@126.com.
